# Interleukin-15 responses to acute and chronic exercise in adults: a systematic review and meta-analysis

**DOI:** 10.3389/fimmu.2023.1288537

**Published:** 2024-01-03

**Authors:** Mousa Khalafi, Aref Habibi Maleki, Michael E. Symonds, Mohammad Hossein Sakhaei, Sara K. Rosenkranz, Mahsa Ehsanifar, Mallikarjuna Korivi, Yubo Liu

**Affiliations:** ^1^Department of Physical Education and Sport Sciences, Faculty of Humanities, University of Kashan, Kashan, Iran; ^2^Department of Exercise Physiology and Corrective Exercises, Faculty of Sport Sciences, Urmia University, Urmia, Iran; ^3^Academic Unit of Population and Lifespan Sciences, Centre for Perinatal Research, School of Medicine, University of Nottingham, Nottingham, United Kingdom; ^4^Department of Exercise Physiology, Faculty of Sport Sciences, University of Guilan, Guilan, Iran; ^5^Department of Kinesiology and Nutrition Sciences, University of Nevada Las Vegas, Las Vegas, NV, United States; ^6^Institute of Human Movement and Sports Engineering, Zhejiang Normal University, Jinhua, Zhejiang, China

**Keywords:** exercise, interleukin 15, body mass index, myokine, inflammation

## Abstract

**Purpose:**

Interlukin-15 (IL-15) is an inflammatory cytokine that plays a vital role in immunology and obesity-associated metabolic syndrome. We performed this systematic review and meta-analysis to investigate whether exercise promotes circulating IL-15 concentrations in adults.

**Methods:**

We searched PubMed, Web of Science, and Scopus from inception to May, 2023 and identified original studies that investigated the effectiveness of acute and/or chronic exercise on serum/plasma IL-15 levels in adults. Standardized mean differences (SMD) and 95% confidence intervals (CI) were calculated using random effect models. Subgroup analyses were performed based on type of exercise, and training status, health status and body mass indexes (BMI) of participants.

**Results:**

Fifteen studies involving 411 participants and 12 studies involving 899 participants were included in the acute and chronic exercise analyses, respectively. Our findings showed that acute exercise increased circulating IL-15 concentrations immediately after exercise compared with baseline [SMD=0.90 (95% CI: 0.47 to 1.32), p=0.001], regardless of exercise type and participants’ training status. Similarly, acute exercise was also associated with increased IL-15 concentrations even one-hour after exercise [SMD=0.50 (95% CI: 0.00 to 0.99), p=0.04]. Nevertheless, chronic exercise did not have a significant effect on IL-15 concentrations [SMD=0.40 (95% CI: -0.08 to 0.88), p=0.10].

**Conclusion:**

Our results confirm that acute exercise is effective in increasing the IL-15 concentrations immediately and one-hour after exercise intervention, and thereby playing a potential role in improving metabolism in adults.

**Systematic review registration:**

https://www.crd.york.ac.uk/prospero/display_record.php?RecordID=445634, identifier CRD42023445634.

## 1 Introduction

Cytokines comprise a large family of polypeptide signaling molecules that are produced by a variety of immune and non-immune cells (1). Cytokines regulate a large number of biological processes, including growth, differentiation, and pro- or anti-inflammatory signaling pathways upon release in response to stimulus (2). Accumulating evidence suggest that skeletal muscle produce and secret several cytokines known as ‘myokines’ which exert endocrine, autocrine or paracrine functions (3–5). Cytokines intrinsically involved in crosstalk between skeletal muscle and other organs, such as adipose tissue, brain, liver, immune system components, as well as within muscle itself (3–5). During exercise, skeletal muscle secreted myokines are involved in lipid and glucose metabolism, chronic low-grade inflammation, skeletal muscle hypertrophy, tumor growth, and cytokine production in tissues and organs (3, 6). Owing to the multi-system involvement of myokines, interventions that can alter circulating myokines could be novel strategies to treat chronic inflammatory diseases and promote health (7, 8).

Interlukin-15 (IL-15) is a 14-15 KDa member of the four α-helix bundle cytokine, with a primary role in the development, survival, and activation of natural killer cells (9). IL-15 is produced by a variety of tissues (skeletal muscle, placenta, heart and kidney) and cell types (epithelial, monocytes and macrophages (9). IL-15 stimulates immune cell responses primarily through specific IL-15 receptor alpha subunits (IL-15Rα), as well as through beta subunits (shared with IL-2) and gamma subunits (shared with IL-2, IL4, IL-7, IL-9 and IL-21) (10, 11). IL-15 that is expressed in skeletal muscle is reported to possess additional non-immune metabolic roles (12–14). Highly expressed IL-15 in skeletal muscle might be a response to muscular contractions during exercise (15). IL-15 promotes myoblast differentiation, skeletal muscle fiber hypertrophy, and inhibits protein degradation (16–18), suggesting a potential target for maintaining of healthy skeletal muscle, and treating muscle wasting disorders (19). In addition, IL-15 also exhibits anti-diabetic and anti-obesity properties, facilitating glucose metabolism in skeletal muscle, modulating adipose tissue deposition and improving insulin sensitivity (20–23). These immuno-metabolic effects of IL-15 suggest it may be a potential therapeutic target for treating chronic metabolic diseases.

Exercise promotes health and can prevent some chronic diseases where there is crosstalk between skeletal muscle and other organs and/or tissues that may act through cytokines (3, 24). Several systematic reviews have suggested that acute and chronic exercise modulate circulating cytokines, including pro- and anti-inflammatory cytokines, such as IL-6, tumor necrosis factor alpha (TNF-α), and IL-10 (25–30). However, currently there is no published comprehensive meta-analysis on IL-15. An increased expression and secretion of IL-15 is one potential mediator of the effect of exercise on skeletal muscle function although the response is inconsistent (31–38). In addition, there are several moderators that affect how acute or chronic exercise influences IL-15 concentrations. The extent to which exercise duration may modify the IL-15 response is unclear. Therefore, we performed this comprehensive meta-analysis to investigate the effect of acute and chronic exercise interventions on circulating IL-15 concentrations in adults.

## 2 Materials and methods

The current systematic review and meta-analysis was conducted following the guidelines of the Preferred Reporting Items for Systematic Reviews and Meta-analyses (PRISMA) (39) and the Cochrane Handbook of Systematic Reviews of Interventions (40). The protocol was registered prospectively with ID: CRD42023445634.

### 2.1 Search strategy

A comprehensive literature search was performed in three main electronic databases including PubMed, Web of Science, and Scopus. Searches were conducted from inception to May 1, 2023 by two independent reviewers (A H M and M H S). The search strategy was performed using the Boolean operators **“**AND**”** and **“**OR**”**, and a combination of the following key words: **“**exercise**”**, **“**exercise training**”**, **“**physical activity**”**, **“**aerobic training**”**, **“**aerobic exercise**”**, **“**resistance training**”**, **“**resistance exercise**”**, **“**combined training**”**, **“**combined exercise**”**, **“**interval training**”**, **“**interval exercise**”**, **“**endurance training**”**, **“**endurance exercise**”**, **“**strength training**”**, **“**strength exercise**”**, **“**IL-15**”**, **“**IL15**”**, **“**Interleukin 15**”**, and Interleukin-15**”**. Additional filters included English language, human participants, and article/document type when they were available in electronic databases. In addition, searches in Google Scholar, as well as manual searches of the reference lists of all included studies, was conducted to obtain any relevant records that may have been missed.

### 2.2 Study eligibility and selection

Studies were eligible for inclusion if they met the following PICO (population, intervention, comparison, and outcome) criteria: (1) For population, human participants with mean age ≥ 18 years, regardless of biological sex, and health and fitness status, were included. Participants were classified into subgroups based on their training status (untrained or trained). For the untrained subgroup, studies recruited participants who had no history of regular exercise training for at least 6 months prior to the study. For the trained and athlete subgroup, studies recruited participants who reported at least five scheduled exercise sessions per week for at least one year. (2) For the intervention, studies with any mode of exercise (acute or chronic), irrespective of type (aerobic, resistance, or combined) were included. For acute exercise, studies with single session of exercise were included. For chronic exercise, studies with exercise duration ≥ 2 weeks were considered to include. Shorter exercise duration was considered in our analysis to distinguish any possible minimal adaptations to exercise. Recent meta-analyses have shown some metabolic adaptations to shorter-term chronic exercise training (41, 42). Nevertheless, the exercise duration in our included trials is five week or more, as there were no eligible studies with shorter exercise duration. There were no limitations for other exercise characteristics, such as intensity, frequency, and time. (3) To investigate the effects of acute exercise, post-exercise values versus pre-exercise values were required. For chronic exercise, the effects of training versus a non-exercise control group were required. (4) For outcomes, studies that reported serum or plasma levels of IL-15, measured using a fully validated method, were included.

Other inclusion criteria were that articles must have been peer-reviewed and published in English. Exclusion criteria were studies with animal models, conference abstracts and non-original studies. Chronic exercise studies with non-randomized trials or studies without control groups were excluded. Two authors (A H M and M H S) independently performed study selection, and any discrepancies between these authors were resolved through consensus with other authors (M Kh and M K). For study selection, all retrieved articles were exported to Endnote (version 20.21). After removing the duplicate articles, the titles and abstracts were screened, followed by full-texts assessment of the remaining articles.

### 2.3 Data extraction and synthesis

The following data were extracted from all included articles: (1) study characteristics. including publication year, and study design (2) characteristics of participants including sample size, biological sex, age, body mass index (BMI), and health and training status, (3) exercise training characteristics i.e. mode, duration, intensity, time, and frequency, and (4) data of outcome variable and assessment methods. To calculate the effect sizes (ES) for acute exercise, means and standard deviations (SD) or mean changes (post-values – pre-values) and their SD, for the exercise group only, for pre-exercise, immediate post-exercise, and one-hour post-exercise outcomes were extracted. To calculate the effect sizes for chronic exercise, means and SDs, or mean changes (post-values – pre-values) and their SDs, for exercise and non-exercise groups were extracted. When required, means and SDs were calculated from standard errors, medians, ranges and IQRs (40, 43, 44). In addition, when required, the data were extracted from figures using Getdata Graph Digitizer. If a study had several exercise arms, all were included. To obtain any missing or additional data from the studies that were published within recent 5 years, we contacted respective corresponding authors. Data extraction was performed independently by two authors (A H M and M H S), and potential discrepancies between the authors were resolved through consensus with other authors (M Kh and M K).

### 2.4 Quality assessment

Study quality was assessed using the Physiotherapy Evidence Database (PEDro) scale, which includes ratings for 11 methodological issues (45). However, two of the items, including blinding of participants, and blinding of intervention providers, were excluded as they are typically not possible in exercise interventions. Finally, study quality was determined using the 9 items listed in **Supplementary Table 1**, rated from 0 (lowest) to 9 (highest). Two authors (A H M and M H S) assessed the quality of included studies, and any disagreements were resolved via consensus with other authors (M Kh and M K).

### 2.5 Statistical analysis

All statistical analyses were performed using version 3 of Comprehensive Meta-analysis Software (CMA3, Biostat Inc., Englewood, NJ, USA). Three separate analyses were performed to calculate standardized mean differences (SMD) and 95% confidence intervals (CIs) using random effects models as follows: 1) effects of acute exercise on immediate post-exercise IL-15 levels using the data from immediately post-exercise versus pre-exercise, 2) effects of acute exercise on IL-15 changes during post-exercise recovery using data from one-hour post-exercise versus pre-exercise, and 3) effects of chronic exercise on IL-15 levels using data from exercise training versus non-exercise control groups. In addition, several sub-group analyses were performed based on types of exercise (aerobic, resistance, and combined training) and training status of participants (un-trained, trained and athletes) for acute exercise analyses. Subgroup analyses for chronic exercise studies, included BMIs (< 30 kg/m^2^ vs. ≥ 30 kg/m^2^), ages (< 50 years vs. ≥ 50 years), health status of participants (without cardio-metabolic diseases vs. with cardio-metabolic diseases), intervention durations (< 16 weeks vs. ≥ 16 weeks), and exercise types (aerobic, resistance, and combined training). Subgroup analyses were performed only when there was enough number of studies for each subgroup (at last 5 trials). The magnitude of effect sizes was interpreted using the Cochrane guidelines: trivial (< 0.2), small (0.2 to < 0.5), moderate (0.5 to 0.8), and large (> 0.8). The I^2^ statistic was used to assess heterogeneity according to the Cochrane guidelines: low (25%), moderate (50%), and high (75%). Publication bias was assessed using visual interpretation of funnel plots and Egger**’**s tests where p values were **<**0.10. To ensure that the results were not influenced by a single study, sensitivity analysis was conducted by omitting individual studies, as well as omitting studies with very large effect sizes. The trim and fill method was used to address any potential effects of publication bias, when present in visual interpretation of funnel plots. -

## 3 Results

### 3.1 Literature search

**Figure 1:**
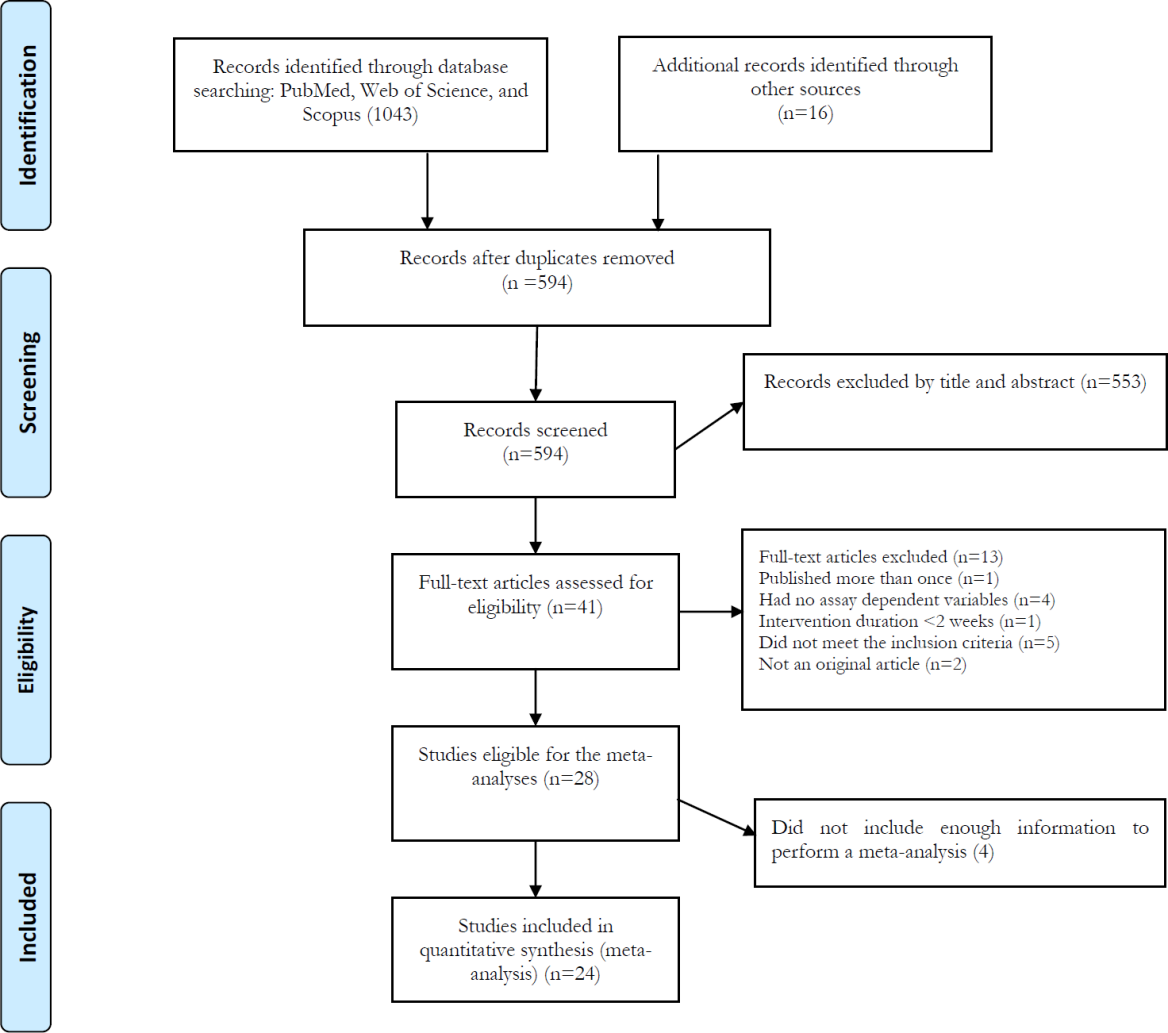
Flow diagram of systematic literature search according to PRISMA.

The initial database searches identified 1,059 records. After removing duplicates, 594 articles remained for screening based on titles and abstracts. After screening the titles and abstracts, 553 irrelevant records were excluded and 41 articles were remained for the full-text assessment. Of 41 articles, 13 were excluded with the reasons presented in **Figure 1**, and 4 articles cannot include in the meta-analysis due to insufficient data. Finally remaining 24 articles were included in the meta-analysis. Among the included studies, 12 studies (31, 31, 46–49, 53–55) investigated the acute exercise effect, nine (58–66) investigated the chronic exercise effect, and three (36, 56, 57) investigated both acute and chronic exercise effects on IL-15.

### 3.2 Participant characteristics

For acute exercise, a total of 411 participants with mean ages and BMIs ranging from ~19 to 84 years and 20 to 35 kg/m^2^, respectively, were included. Of the 15 included studies, four studies consisted of females (31, 51, 56, 57), six studies consisted of males (36, 46, 48, 50, 52, 54), and the remaining studies consisted of both males and females (47, 49, 55). Two studies did not clearly report the biological sex of participants (35, 53). For chronic exercise, 899 participants with mean ages ranging from ~37 to 90 years, and BMIs ranging from 23 to 35 kg/m^2^ were included. Among the 12 studies, seven studies included females (56–58, 61, 63–65), two studies included males (36, 60), and three studies included both males and females (59, 62, 66). Participants had a wide range of health conditions and diseases, such as overweight, obesity, diabetes, and high risk of breast cancer.

### 3.3 Exercise characteristics

For the acute exercise protocol, aerobic or resistance exercise were used in majority of the studies, while maximal incremental, submaximal exercise tests and marathon running were used in other studies. Exercise duration was ranged from 3 to 360 minutes, while the intensity was widely differ for each mode of exercise. For resistance exercise, the intensity was ranged from 30 to 80% 1RM, and for aerobic exercise the intensity was ranged from 50 to 70% HR_max_. In addition, long-distance trail running or standard exercise test were performed in several studies. For chronic exercise studies, trials used aerobic, resistance, combined and sprint, and high intensity interval trainings. Briefly, for chronic exercise, intervention duration was ranged from 5 weeks to 6 months, and weekly exercise sessions ranged from 1–3 to 5 sessions. Most of the chronic exercise was performed under supervision. Full details of exercise intensity and duration with the mode of exercise are summarized in **Tables 1**, **2**.

**Table 1:** Summary of participant and intervention characteristics of acute exercise.

Study details		Participant characteristics					Exercise characteristics		
Author and Year of publication	Allocation	*n* (sex)	Health status	Training status	Age (years)	BMI (kg/m^2^)	Exercise mode	Exercise protocol	Time of measurements
Bugera et al, 2018 (46)	Randomized crossover	10 (M)	Healthy	Trained	25.8 ± 3.6	25.9 ± 2.2	EX_1_: Resistance with low load EX_2_: Resistance with high load	EX_1_: 4 sets of 15-30 reps at 30% 1RM EX_2_: 4 sets of 7 reps at 80% 1RM	Pre, Post, 1h Post
Christiansen et al, 2013 (47)	Single intervention	15 (M & F)	Healthy	Untrained	32.7 ± 12.0	22.4 ± 2.0	Aerobic	120 min ergometer bicycling at 55-60% of HR_max_	Pre, Post
		16 (M & F)	Overweight & obese	Untrained	41.3 ± 4.0	31.8 ± 3.0			
Eskandari et al, 2020 (48)	Single intervention	10 (M)	Healthy	Untrained	26.0 ± 3.0	24.2 ± 2.1	Resistance	Whole body resistance exercises; 3 sets with 8-10 reps at 70% 1RM	Pre, Post, 1h Post
Garneau et al, 2020 (31)	Single intervention	5 (F)	Overweight	Untrained	28.8 ± 8.4	27.8 ± 1.4	Aerobic	60 min at 60% VO_2peak_	Pre, Post, 1h Post
		6 (F)	Obese	Untrained	30.0 ± 6.9	35.4 ± 5.4			
Hingorjo et al, 2018 (49)	Single intervention	75 (M & F)	Healthy	Untrained	19.4 ± 1.1	19.6 ± 2.0	Aerobic	Queen’s College Step Test	Pre, Post
		58 (M & F)	Overweight	Untrained	19.4 ± 1.0	27.7 ± 4.2			
Kapilevich et al, 2017 (50)	Random (untrained groups)	10 (M)	Healthy	Athletes	19.9 ± 1.4	ND	Resistance	Static load under the knee (weight amount: 50% 1RM; deadlift)	Pre, Post, 1h Post
		5 (M)	Healthy	Untrained	19.5 ± 0.7				
		10 (M)	Healthy	Athletes	20.8 ± 1.4		Aerobic	PWC170 standard test includes pedaling at two different power levels	
		5 (M)	Healthy	Untrained	20.2 ± 1.1				
Luk et al, 2021 (51)	Crossover	13 (F)	Healthy	Trained	24.0 ± 14.4	25.0 ± 11.5	Resistance	traditional sets (4x10, 120-s inter- set rest), 70% 1RM	Pre, Post, 1h Post
				Trained				rest-redistribution (4x 2x5) with 30-s intra- set rest after 5 repetitions and 90-s inter- set rest, 70% 1RM	
Marcucci-Barbosa et al, 2020 (52)	Single intervention	9 (M)	Healthy	Athletes	32.2 ± 10.2	25.9 ± 3.2	Aerobic	10 km running race Experimental trials involving 10 km of running at one’s best possible performance	Pre, Post
Minuzzi et al, 2019 (53)	Single intervention	9 (ND)	Healthy	Untrained	31.8 ± 3.0	21.8 ± 2.0	Aerobic	Maximal incremental test on a cycle ergometer started at 75W for a 3 min followed by 25W increments every 3 min until volitional exhaustion	Pre, Post, 1h Post
		10 (ND)	Healthy	Untrained	54.2 ± 5.9	24.3 ± 3.2			
		20 (ND)	Healthy	Athletes	53.1 ± 8.8	25.1 ± 4.6			
Pérez-López et al, 2018 (35)	Single intervention	14 (ND)	Healthy	Untrained	24.9 ± 4.8	25.6 ± 3.1	Resistance	Lower-body exercises; 4 sets with 8-12 reps at 75% 1RM	Pre, Post, 1h Post
Rinnov et al, 2014 (36)	Single intervention	8 (M)	Healthy	Trained	27.0 ± 3.7	23.3 ± 1.9	Aerobic	3 hours cycling at 60% VO_2max_	Pre, Post
Tamura et al, 2011 (54)	Single intervention	13 (M)	Healthy	Untrained	28.1 ± 4.7	22.4 ± 2.5	Aerobic	30 min at 70% HR_max_	Pre, Post, 1h Post
Yargic et al, 2019 (55)	Single intervention	37 (M & F)	Healthy	Athletes	39.0 ± 10.1	23.2 ± 2.2	Aerobic	long-distance trail running (35 km race run, minimum 6 hours)	Pre, Post
Micielska et al, 2019 (56)	Single intervention	33 (F)	Healthy	Untrained	40.0 ± 11.0	ND	Resistance	High-intensity circuit exercises with body weight for 3 circuit at 80-90% HR_max_	Pre, 1h Post
Urzi et al, 2019 (57)	Single intervention	20 (F)	Healthy older	Untrained	84.4 ± 7.7	28.0 ± 5.5	Resistance	50 min whole body elastic resistance training at a rating of 11-15 BRPE	Pre, Post

Ex, exercise; CON, control; F, female, M, male; HIIT, high-intensity interval training; VO_2max_, peak maximal or peak oxygen uptake; HR_max_, peak maximal or peak heart rate; HRR, heart rate reverse; reps repetitions; 1RM, one-repetition maximum; BRPE, borg rating of perceived exertion; ND, not-described.

**Table 2:** Summary of participant and intervention characteristics of chronic exercise.

Study details		Participant charecteristics					Exercise charecteristics (Chronic)		
Author and Year of publication	Allocation	n (sex)	Health Status	Training status	Age (years)	BMI (kg/m^2^)	Type	Trinining protocol	Duration (weekly sessions)
Banitalebi et al, 2019 (58)	Random	52 (F)	Overweight with T2D	Untrained	EX_1_:55.4 ± 5.9 EX_2_:54.1 ± 5.4 CON:55.7 ± 6.4	EX_1_:29.3 ± 3.0 EX_2_:28.7 ± 4.3 CON:30.1 ± 3.5	EX_1_: Sprint interval EX_2_: Combined	EX_1_: 4 sets of 30 second at maximum ability. EX_2_: combined training including 30 min aerobic training at 60-70% HR_max_ and whole-body resistance exercise including 1-3 sets with 10-15 reps and with 10-15 RM	10 weeks (3 Supervised)
Beavers et al, 2010 (59)	Random	372 (M & F)	Healthy older	Untrained	EX:76.4 ± 4.1 CON:77.0 ± 4.4	EX:30.7 ± 6.0 CON:29.8 ± 5.5	Combined	Combined training including 40-60 min of aerobic, resistance, balance & flexibility training, aerobic at 11-15 BRPE, and lower body resistance training at 15-16 BRPE	12 months (1-3 Supervised & Unsupervised)
Brunelli et al, 2015 (60)	Random	54 (M)	Obese	Untrained	EX:49.3 ± 5.36 CON:48.0 ± 6.1	EX:31.0 ± 1.7 CON:31.0 ± 1.5	Combined	Combined training including 30 min aerobic training at 50-85% VO_2peak_ and 30_ min_ resistance training inclduing 3 sets at 6-10 RM	24 weeks (3 Supervised)
Coletta et al, 2021 (61)	Random	33 (F)	Overweight or obese pre–post menopause (High risk for breast cancer)	Untrained	EX_1_:64.4 ± 6.2 EX_2_:66.1 ± 13.6 CON:62.6 ± 7.0	EX_1_:32.3 ± 8.6 EX_2_:32.0 ± 5.6 CON:29.9 ± 2.5	EX_1_: HIIT EX_2_: Aerobic	EX_1_: 4 sets of 4 min at 90-100% HR_peak_ and 3 min active rest (total time: 33 min) EX_2_: 41 min aerobic training at 60-70% HR_peak_	12 weeks (3 Supervised)
Corrêa et al, 2021 (62)	Random	97 (M & F)	Chronic kidney disease	Untrained	EX:58.0 ± 6.0 CON:58.0 ± 5.0	EX:33.6 ± 2.0 CON:33.2 ± 1.6	Resistance	1-3 sets of 8-12 reps exercise at 50-70% 1RM	6 months (3 Supervised)
Nikseresht et al, 2022 (63)	Random	41 (F)	Overweight	Untrained	EX_1_:37.2 ± 4.7 EX_2_:37.2 ± 4.7 CON:37.2 ± 4.7	EX_1_:29.5 ± 1.5 EX_2_:29.9 ± 2.0 CON:29.7 ± 2.1	EX_1_: Resistance with linear periodized EX_2_: Resistance with flexible non-linear periodized	EX_1_: week 1-4: 2 sets, 12-15RM; week 5-8: 3 sets, 8-10RM; week 9-12: 4 sets, 3-5RM Ex_2_: week 1-2: 2 sets, 12-15RM; week 3-4: 3 sets, 8-10RM; week 5-6: 4 sets, 3-5RM; week 7-12: First day: 4 sets, 3-5RM; Second day: 3 sets, 8-10RM; Third day: 2 sets, 12-15RM	12 weeks (3 Supervised)
Nishida et al, 2015 (64)	Random	69 (F)	Healthy older	Untrained	EX:70.4 ± 5.8 CON:69.7 ± 6.6	EX:24.2 ± 3.7 CON:22.5 ± 2.5	Aerobic	10-20 min home-based exercise with steps at lactate threshold intensity	12 weeks (3 Unsupervised)
Pérez-López et al, 2022 (65)	Random	47 (F)	Obese ppre–post menopause	Untrained	EX_1_:58.7 ± 2.9 EX_2_:56.7 ± 3.7 EX_3_:43.1 ± 2.8 CON:56.9 ± 5.8	EX_1_:33.8 ± 5.3 EX_2_:32.9 ± 4.2 EX_3_:37.0 ± 2.8 CON:34.9 ± 4.6	EX_1_: Combined EX_2_: Aerobic for post-menopause EX_3_: Aerobic for pre menopause	EX_1_: 20 min aerobic training at 55-75% HRR, and 20 min resistance training including 3 sets with 8-12 reps at 65% 1RM EX_2_ and EX_3_: 60 min at 55-75% HRR	12 weeks (3 Supervised)
Rinnov et al, 2014 (36)	Non- random	15 (M)	Healthy	Trained	EX:30.5 ± 5.5 CON:25.2 ± 3.3	EX:25.1 ± 2.1 CON:23.2 ± 2.4	Aerobic	The training consisted of intervals cyclying including 10 sets of 3 min at 85% of maximum power by 3 min of recovery with 40% of maximum power and continuous cycling including 60 min at 60% maximum power.	12 weeks (5 ND)
Tsai et al, 2019 (66)	Random	66 (M & F)	Older with mild cognitive impairment	Untrained	EX_1_:66.0 ± 7.7 EX_2_:65.4 ± 6.8 CON:65.2 ± 7.0	EX_1_:23.5 ± 3.3 EX_2_:24.4 ± 3.1 CON:23.4 ± 2.8	EX_1_: Aerobic EX_2_: Resistance	EX_1_: 30 min at 70-75% HRR EX_2_: whole body resitace exercise with 3 sets of 10 reps at 75% 1RM	16 weeks (3 Supervised)
Micielska et al, 2019 (56)	Non- random	33 (F)	Healthy	Untrained	EX:40.0 ± 11.0 CON:45.0 ± 13.0	ND	Resistance	High-intensity circuit training including 9 exercises with body weight for 3 circuit at 80-90% HR_max_	5 weeks (3 Supervised & Unsupervised)
Urzi et al, 2019 (57)	Random	20 (F)	Healthy older	Untrained	EX:84.4 ± 7.7 CON:88.9 ± 5.3	EX:28.0 ± 5.5 CON:29.1 ± 5.1	Resistance	50 min whole body elastic resistance training at a rating of 11-15 BRPE	12 weeks (3 Supervised)

Ex, exercise; CON, control; F, female, M, male; HIIT, high-intensity interval training; VO_2max_, peak maximal or peak oxygen uptake; HR_max_, peak maximal or peak heart rate; HRR, heart rate reverse; reps, repetitions; 1RM, one-repetition maximum; BRPE, borg rating of perceived exertion; ND, not-described.

### 3.4 Meta-analysis

#### 3.4.1 Effects of acute exercise on immediate post-exercise IL-15

**Figure 2:**
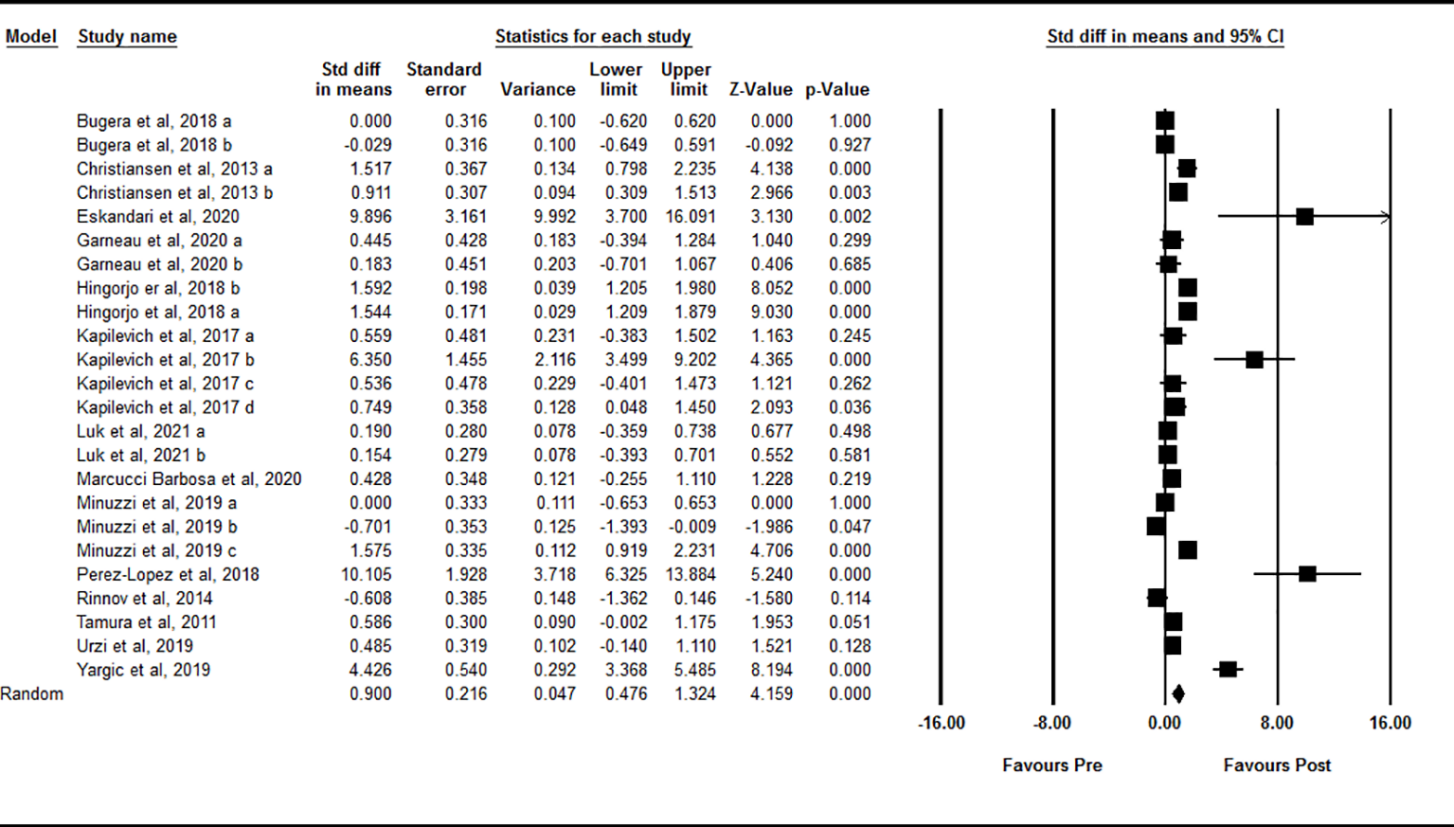
Forest plot of the effects of acute exercise on immediate post-exercise IL-15. Data are reported as SMD (95% confidence limits). SMD, standardized mean difference.

Based on 24 intervention arms, acute exercise significantly increased circulating IL-15 levels immediately after exercise compared with baseline [SMD=0.90 (95% CI: 0.47 to 1.32), p=0.001] (**Figure 2**). There was significant and high heterogeneity among the included studies (I^2 ^= 88.60, p=0.001). Visual interpretation of the funnel plots suggested publication bias, but the Egger’s test did not (p=0.40). The Trim and fill method identified four missing studies from the right side of the plots. When accounting for these missing studies, the overall effect size was [SMD=1.21 (95% CI: 0.77 to 1.64)]. Sensitivity analysis by omitting individual studies did not change the significance or the direction of the effect. In addition, sensitivity analysis by omitting studies with very large effect size did not change the significance or direction of any effect. Subgroup analysis based on type of exercise showed that both aerobic [SMD=0.86 (95% CI: 0.37 to 1.34), p=0.001] and resistance [SMD=1.05 (95% CI: 0.23 to 1.79), p=0.01] exercise led to increased circulating IL-15 immediately following acute exercise. In addition, subgroup analysis based on training status of participants showed that acute exercise led to increased circulating IL-15 in both athletes [SMD=2.32 (95% CI: 0.86 to 3.78), p=0.001] and untrained [SMD=0.86 (95% CI: 0.35 to 1.37), p=0.001] individuals, but not in trained individuals [SMD=0.00 (95% CI: -0.27 to 0.27), p=1.00] immediately after exercise.

#### 3.4.2 Effects of acute exercise on IL-15 during post-exercise recovery

**Figure 3:**
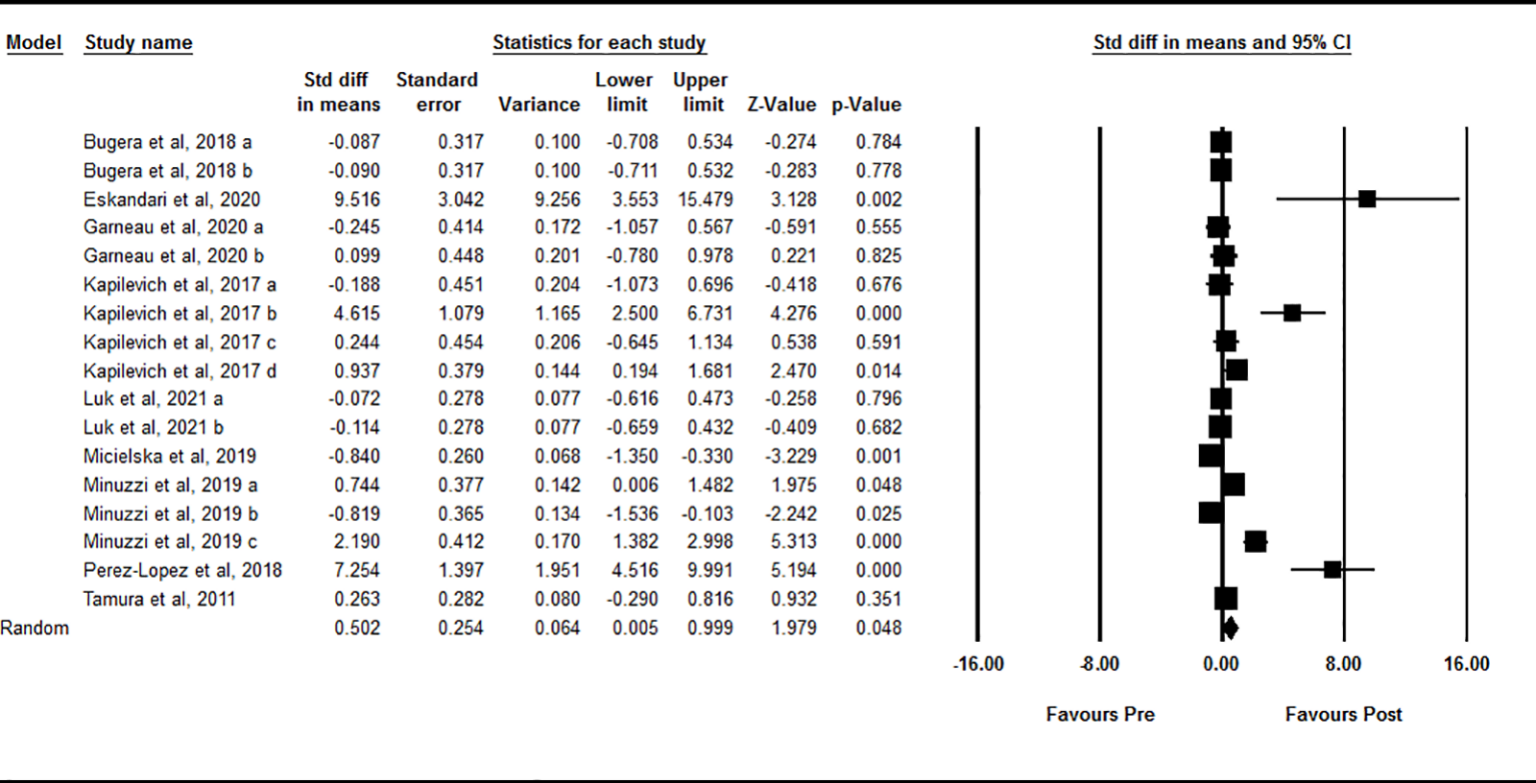
Forest plot of the effects of acute exercise on one-hour post-exercise IL-15. Data are reported as SMD (95% confidence limits). SMD, standardized mean difference.

Data from 17 intervention arms showed that acute exercise significantly increased circulating IL-15 at one-hour after exercise compared with baseline [SMD=0.50 (95% CI: 0.00 to 0.99), p=0.04] (**Figure 3**). We found a significant and high heterogeneity among included studies (I^2 = ^85.37, p=0.001). Visual interpretation of the funnel plots and the Egger’s test revealed publication bias (p=0.001). The Trim and fill method identified one missing study from the right side of the plots. When accounting for this missing study, the overall effect size was [SMD=0.66 (95% CI: 0.11 to 1.20)]. Sensitivity analysis by omitting individual studies changed the significance. In addition, sensitivity of analysis by omitting studies with very large effect size changed the significance [SMD=0.12 (95% CI: -0.25 to 0.49), p=0.52]. Subgroup analysis based on type of acute exercise showed that both aerobic [SMD=0.42 (95% CI: -0.18 to 1.02), p=0.17] and resistance [SMD=0.68 (95% CI: -0.10 to 1.46), p=0.09] exercise increased circulating IL-15, but the change was not statistically significant. Subgroup analysis for the training status of participants revealed that increased circulating IL-15 at one-hour during post-exercise was seen only in athletes [SMD=2.27 (95% CI: 0.75 to 3.79), p=0.003], but not in untrained [SMD=0.33 (95% CI: -0.35 to 1.02), p=0.34] and trained [SMD=-0.09 (95% CI: -0.38 to 0.19), p=0.53] individuals.

#### 3.4.3 Effects of chronic exercise on IL-15

**Figure 4:**
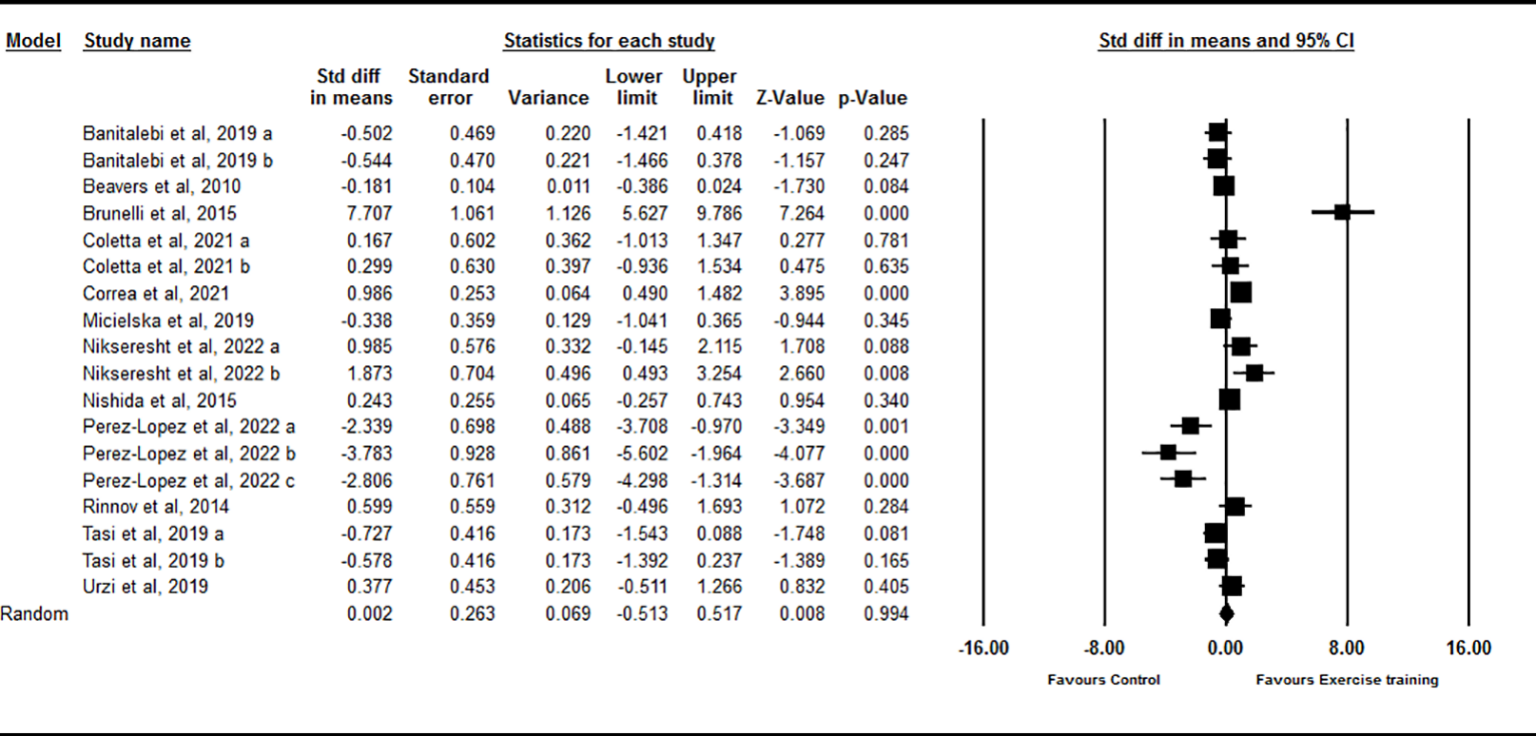
Forest plot of the effects of chronic exercise on IL-15. Data are reported as SMD (95% confidence limits). SMD, standardized mean difference.

Based on 18 intervention arms, chronic exercise did not significantly change circulating IL-15 compared with control [SMD=0.002 (95% CI: -0.51 to 0.51), p=0.99] (**Figure 4**). There was significant and high heterogeneity among included studies (I^2 ^= 87.25, p=0.001). Visual interpretation of the funnel plots suggested publication bias, but the Egger’s test did not showed the bias (p=0.79). The Trim and fill method identified five missing studies from the right side of the plots. When accounting for these missing studies, the overall effect size increased [SMD=0.65 (95% CI: 0.08 to 1.21)]. Sensitivity analysis by omitting individual studies did not change the significance. Subgroup analysis based on type of exercise training showed that chronic aerobic [SMD=-0.63 (95% CI: -1.42 to 0.15), p=0.11], resistance [SMD=0.46 (95% CI: -0.21 to 1.15), p=0.18], and combined [SMD=0.91 (95% CI: -1.24 to 1.46), p=0.40] exercise training did not significantly change circulating IL-15. Subgroup analysis based on the health status of participants showed that exercise training did not significantly change circulating IL-15 in healthy individuals [SMD=-0.02 (95% CI: -0.69 to 0.64), p=0.93] or participants with chronic diseases [SMD=0.12 (95% CI: -0.60 to 0.85), p=0.74]. Subgroup analysis based on BMI showed that chronic exercise training did not significantly change circulating IL-15 in individuals with [SMD=-0.08 (95% CI: -1.23 to 1.07), p=0.88] or without [SMD=0.08 (95% CI: -0.38 to 0.55), p=0.71] obesity. Subgroup analysis based on the intervention duration showed that exercise training did not significantly change circulating IL-15 with medium-term (durations < 16 weeks) [SMD=-0.32 (95% CI: -0.93 to 0.28), p=0.29] or long-term (durations ≥ 16 weeks) [SMD=0.93 (95% CI: -0.23 to 2.09), p=0.11] interventions.

### 3.5 Quality assessment

The overall quality of the included studies is summarized in **Supplementary Table 1**. For acute exercise, the PEDro scores ranged from three to seven, out of a possible score of nine. For chronic exercise, the PEDro scores ranged from five to seven, out of a possible score of nine.

## 4 Discussion

Our findings demonstrated that acute exercise increased circulating IL-15 concentration immediately after exercise, and this response was independent of exercise type, but appears to be modulated by the training status of participants. However, the increased IL-15 at one-hour post-exercise was inconsistent with additional sensitivity analyses were required to show the significance and effect size. Chronic exercise was not effective in altering the circulating IL-15. These findings suggest that increased IL-15 is a transient response to exercise in untrained and trained individuals. Although we confirmed increased IL-15 concentrations immediately after exercise, the potential mechanism is not clear given that secretion of IL-15 occurs from several tissues, and the regulation of IL-15 occurs by several receptor isoforms (56, 67). Skeletal muscle may be the main source for exercise-induced circulating IL-15 (32, 33) for which a positive correlation has been shown with the protein content of muscle (35). Exercise promotes gene expression of IL-15 in human skeletal muscle, but it is unclear whether it translates to increase circulating IL-15 concentrations (68–70). The secretion of IL-15 from muscle is not necessarily dependent on transcriptional changes in its mRNA, and the intramuscular pool of secretable IL-15 could be the primary source of IL-15 following exercise (32, 35, 71). It is considered that IL-15 is a promising target in the treatment of metabolic disorders through improvements in lipid and glucose metabolism, and pharmacologically beneficial in cancer immunotherapy through anti-tumor activity (20, 72, 73). Since IL-15 is a myokine with significant metabolic, anabolic, and immune functions (20, 74), exercise-induced changes might be beneficial for prevention and management of metabolic disorders in humans.

Based on our analyses, IL-15 increases immediately after exercise, with an inconsistent increase during the recovery period (within one-hour), that is not unexpected given the relatively short half-life of IL-15 (about 30 min) (15, 31). In addition, the effect size was larger in athletes compared to trained and untrained individuals, and was significant only in athletes at one-hour post-exercise. This larger effect size may be due to an adaption to chronic training, which leads to increased expression of IL-15 and accumulation in skeletal muscle potentially enhancing its secretion (35, 36, 53). Previous human studies have shown that acute exercise can elevate circulating IL-15 concentrations in 10 min after exercise and it may sustain until 120 min following exercise (32, 54). Nevertheless, the increased IL-15 concentrations subsequently returned to pre-exercise level at 3-h during post-exercise recovery (54). We further demonstrated that exercise increased circulating IL-15 regardless of exercise type. It appears that both aerobic and resistance exercise, lead to stimulate IL-15 secretion through skeletal muscle contraction and metabolic training adaptations, particularly in the immediate post-exercise period.

Several meta-analyses have provided evidence that chronic exercise can modulate circulating cytokines (30, 75, 76), further studies are required to determine whether exercise training leads to adaptations in circulating IL-15. Inconsistent reports have shown increased, decreased and non-significant effects on IL-15 following chronic exercise training (26, 57–66). Our findings also showed that chronic exercise had no effect on circulating IL-15. It is worth noting that subgroup analyses indicated variable effect sizes according to exercise type. Effect sizes were negative, medium, and non-significant with aerobic training, but positive, small and non-significant with resistance training, and large with combined training, suggesting that exercise type may contribute to heterogeneity and introduce bias in interpreting the results. IL-15 is an anabolic growth factor that is expressed in skeletal muscle and acts in an endocrine fashion (18), that may be dependent on acute changes (38). Furthermore, chronic exercise may increase the intramuscular supply of IL-15, thereby contributing to the larger effect sizes in trained participants following acute exercise. In addition, we showed large and medium effect sizes following resistance and combined training, indicating that the higher circulating values of IL-15 could be a result of training adaptations as compared to aerobic exercise training. Subgroup analyses were also performed based on the health status and BMI of participants with chronic exercise training, and results indicated that these factors had no effect on IL-15.

The current meta-analysis has several limitations that should be considered when interpreting our results. For acute exercise, we included data at the one-hour time point after exercise, and the available data did not allow for closer examination of longer responses. There was significant heterogeneity indicated in all of the primary analyses, which may be partly explained by exercise type and training status of participants. Additionally, publication bias was detected with visual interpretation of funnel plots, but the Trim and fill method confirmed that adding missing studies either did not change the effect sizes, or increased them. In addition, the variation in exercise protocols (acute or chronic), lack of adequate information provided in some studies, and reporting of exercise intensity using different criteria (such as the percentage of 1RM, or 10-RM, HRmax, VO_2_max, BRPE), did not allow us to perform sub-group analyses based on exercise characteristics, including intensity and duration. These issues should be considered in future studies to address the effect of exercise on IL-15 concentrations.

## 5 Conclusions

Our systematic review and meta-analysis demonstrated that acute, but not chronic exercise training, increased circulating IL-15 concentrations immediately after exercise. Chronic exercise may increase IL-15 muscle content, leading to an acute increase in circulating IL-15 concentrations in subsequent periods of exercise.

## Data availability statement

The original contributions presented in the study are included in the article/**Supplementary Material**. Further inquiries can be directed to the corresponding authors.

## Author contributions

MKh: Conceptualization, Data curation, Investigation, Methodology, Software, Supervision, Validation, Visualization, Writing – original draft, Writing – review & editing. AM: Data curation, Investigation, Methodology, Software, Writing – original draft. MES: Conceptualization, Investigation, Methodology, Supervision, Validation, Writing – review & editing. MHS: Conceptualization, Data curation, Investigation, Methodology, Software, Validation, Visualization, Writing – original draft. SR: Conceptualization, Data curation, Investigation, Methodology, Software, Validation, Writing – review & editing. ME: Conceptualization, Data curation, Investigation, Methodology, Software, Writing – original draft. MKo: Conceptualization, Data curation, Funding acquisition, Investigation, Methodology, Validation, Writing – original draft, Writing – review & editing. YL: Conceptualization, Funding acquisition, Investigation, Methodology, Validation, Writing – original draft, Writing – review & editing.
