# Supplementary material for: Interleukin-15 responses to acute and chronic exercise in adults: a systematic review and meta-analysis
Source: Front Immunol. 2024 Jan 3;14:1288537. doi: 10.3389/fimmu.2023.1288537 (PMC10791876; doi:10.3389/fimmu.2023.1288537)
Supplement: Supplementary file 1 [file Table_1.docx]

**Supplementary Table 1.** Risk of bias assessment

| **Authors & Year** | **Criteria 1** | **Criteria 2** | **Criteria 3** | **Criteria 4** | **Criteria 5** | **Criteria 6** | **Criteria 7** | **Criteria 8** | **Criteria 9** | **total** |
| --- | --- | --- | --- | --- | --- | --- | --- | --- | --- | --- |
| **Chronic interventions** | | | | | | | | | | |
| Banitalebi et al., 2019 [54] | ✓ | ✓ | ✓ | ✓ | ✓ | x | x | ✓ | ✓ | 7 |
| Beavers et al., 2010 [55] | ✓ | ✓ | ? | ✓ | ✓ | ✓ | x | ✓ | ✓ | 7 |
| Brunelli et al., 2015 [56] | ✓ | ✓ | ? | ✓ | ? | ✓ | x | ✓ | ✓ | 6 |
| Coletta et al., 2021 [57] | ✓ | ✓ | ? | ✓ | ? | ✓ | ✓ | ✓ | ✓ | 7 |
| Corrêa et al., 2021 [58] | ✓ | ✓ | x | ✓ | x | ✓ | x | ✓ | ✓ | 6 |
| Nikseresht et al., 2022 [59] | ✓ | ✓ | x | ? | x | ✓ | x | ✓ | ✓ | 5 |
| Nishida et al., 2015 [60] | ✓ | ✓ | ? | ✓ | x | ✓ | x | ✓ | ✓ | 6 |
| Pérez-López et al., 2022 [61] | ✓ | ✓ | ✓ | ✓ | x | ✓ | x | ✓ | ✓ | 7 |
| Rinnov et al., 2014 [49] | ✓ | ? | ? | ✓ | x | ✓ | x | ✓ | ✓ | 5 |
| Tsai et al., 2019 [62] | ✓ | ✓ | ✓ | ✓ | x | x | x | ✓ | ✓ | 6 |
| Micielska et al., 2019 [52] | x | x | x | ✓ | x | ✓ | x | ✓ | ✓ | 4 |
| Urzi et al., 2019 [53] | ✓ | ✓ | ? | ✓ | x | ✓ | x | ✓ | ✓ | 6 |
| **Acute interventions** | | | | | | | | | | |
| Bugera et al., 2018 [39] | ✓ | ✓ | ✓ | ✓ | x | ✓ | x | ✓ | ✓ | 7 |
| Christiansen et al., 2013 [40] | ✓ | x | x | x | x | ✓ | x | ✓ | ✓ | 4 |
| Eskandari et al., 2020 [41] | ✓ | ✓ | ✓ | ✓ | x | ✓ | x | ✓ | ✓ | 7 |
| Garneau et al., 2020 [42] | ✓ | ✓ | x | ✓ | x | ✓ | x | ✓ | ✓ | 6 |
| Hingorjo et al., 2018 [43] | ✓ | ✓ | x | ✓ | x | ✓ | x | ✓ | ✓ | 6 |
| Kapilevich et al., 2017 [44] | ✓ | ✓ | x | ✓ | x | ✓ | x | ✓ | ✓ | 6 |
| Luk et al., 2021 [45] | ✓ | x | x | ✓ | x | ✓ | x | x | ✓ | 4 |
| Marcucci-Barbosa et al., 2020 [46] | ✓ | x | x | ✓ | x | ✓ | x | x | ✓ | 4 |
| Minuzzi et al, 2019 [47] | ✓ | ✓ | x | ✓ | x | ✓ | x | ✓ | ✓ | 6 |
| Pérez-López et al., 2018 [48] | x | ✓ | ? | ✓ | x | ✓ | x | ✓ | ✓ | 5 |
| Rinnov et al., 2014 [49] | ✓ | ? | ? | ✓ | x | ✓ | x | ✓ | ✓ | 5 |
| Tamura et al., 2011 [50] | ✓ | x | x | ? | x | ✓ | x | x | ✓ | 3 |
| Yargic et al., 2019 [51] | x | x | x | ✓ | x | ✓ | x | x | ✓ | 3 |
| Micielska et al., 2019 [52] | x | x | x | ✓ | x | ✓ | x | ✓ | ✓ | 4 |
| Urzi et al, 2019 [53] | ✓ | ✓ | ? | ✓ | x | ✓ | x | ✓ | ✓ | 6 |

(1) Eligibility Criteria specified, (2) Random allocation of participants, (3) Allocation concealed, (4) Groups similar at baseline, (5) Assessors blinded, (6) Outcome measures assessed in 85% of participants, (7) Intention to treat analysis, (8) Reporting of between group statistical comparison, (9) Point measures and measures of variability reported for main effects. ‘low (✓), ‘high (x) and unclear (?)
